# The association between dietary sodium density and Hashimoto’s thyroiditis in US adults

**DOI:** 10.3389/fnut.2025.1508195

**Published:** 2025-01-22

**Authors:** Peilin An, Silin Wang, Lingyun Liu, Xuelin Li, Xin Lv

**Affiliations:** ^1^Department of Health Management Center, Chongqing General Hospital, Chongqing University, Chongqing, China; ^2^Department of Electrical Engineering, Chalmers University of Technology, Gothenburg, Sweden; ^3^Department of Clinical Nutrition, The Affiliated Hospital of Yangzhou University, Yangzhou University, Yangzhou, China; ^4^Yangzhou Key Laboratory of Preventive and Translational Medicine for Geriatric Disease (Frailty), The Affiliated Hospital of Yangzhou University, Yangzhou, China

**Keywords:** Hashimoto’s thyroiditis, dietary sodium density, thyroid peroxidase antibody, thyroglobulin antibody, sodium

## Abstract

**Background:**

Hashimoto’s thyroiditis (HT) is an autoimmune thyroid disease characterized by the presence of antibodies against thyroid-specific antigens. Dietary sodium intake has been implicated in the development of several autoimmune diseases, but its association with HT remains unclear.

**Methods:**

This study investigates the relationship between dietary sodium density (the ratio of sodium to energy intake) and HT using data from the National Health and Nutrition Examination Survey (NHANES) from 2007 to 2012. A cross-sectional study was conducted using NHANES data, focusing on adults aged 20 years and older with available dietary and thyroid profile data. Sodium density was calculated from two 24-h dietary recall interviews. Logistic regression models were used to evaluate the associations of sodium density with HT, thyroid peroxidase antibody (TPOAb) and thyroglobulin antibody (TgAb). Restricted cubic spline (RCS) analyses were performed to explore non-linear relationships.

**Results:**

A total of 6,258 participants were included, with 576 (9.20%) diagnosed with HT. An additional unit of dietary sodium density was associated with a 24% increased risk of HT (OR 1.24, 95% CI 1.01–1.50) in adjusted model. A breakpoint at 2.43 mg/kcal in dietary sodium density was identified using a piecewise regression model. Below this threshold, HT risk increased with rising sodium density, while above it, the risk plateaued. Higher sodium density was also associated with increased TPOAb positivity (OR 1.28, 95% CI 1.05–1.56), but not TgAb positivity.

**Conclusion:**

Elevated dietary sodium density is associated with an increased risk of HT and TPOAb positivity, suggesting that sodium intake may play a role in the pathogenesis of HT.

## 1 Introduction

Hashimoto’s thyroiditis (HT), also known as chronic lymphocytic or autoimmune thyroiditis, is an autoimmune thyroid disease (AITD) characterized by increased thyroid volume, lymphocytic infiltration of thyroid tissue, and the presence of antibodies against thyroid-specific antigens (TPOAb, i.e., antibodies against thyroid peroxidase and TgAb, i.e., antibodies against thyroglobulin) (1, 2). Clinically, HT primarily manifests as systemic symptoms resulting from thyroid gland damage, ultimately leading to primary hypothyroidism (1). HT is one of the most common thyroid diseases, with an incidence of 0.3–1.5 cases per 1,000 people per year (3). It is predominantly diagnosed in women (4–10 times more frequently than men) and is more prevalent among older individuals (2, 3). The development of HT is believed to stem from a complex interplay of genetic, environmental, and epigenetic factors (1, 3). Given that the rapid increase in HT incidence cannot be attributed to genetic changes, environmental factors have gained renewed attention. Several environmental triggers, such as increased dietary iodine intake, pollution, psychological stress, smoking, and poor dietary habits, possibly contribute to the development of HT (3, 4). Among these, dietary factors such as the Western diet—high in processed foods, red meat, high-fat dairy, and low fiber—may elevate the risk of HT by promoting inflammation and disrupting gut microbiota (5–7).

Sodium, a major constituent of dietary salt, is crucial for maintaining extracellular fluid volume and cellular membrane potential in mammals. However, excessive sodium consumption has been linked to various non-communicable diseases, including hypertension and cardiovascular disease, which collectively increase overall mortality (8). The World Health Organization (WHO) currently recommends reducing sodium intake to less than 2 g/day (equivalent to 5 g/day of salt) (8). Recent studies have suggested that high sodium intake may also play a role in the development of autoimmune diseases, by reversing the suppressive effects of Regulatory T cells (Tregs) and promoting a shift toward T-helper (Th)-1 and Th17 pro-inflammatory phenotypes (9). Excessive sodium intake has been shown to exacerbate pro-inflammatory responses in patients with rheumatoid arthritis, systemic lupus erythematosus (SLE), multiple sclerosis, and inflammatory bowel disease in animal studies (10–12). However, few studies have explored the relationship between sodium intake and HT, and the role of dietary sodium in HT remains unknown.

Sodium density, defined as the ratio of sodium to energy in the diet, is a useful measure in nutritional epidemiology as it reduces for inter-individual variation in factors (which are not confounders) like body composition (13, 14). Given the strong correlation between sodium and energy intake (15), the relationship between sodium and HT may be influenced by energy intake levels (13). Therefore, in this study, we used sodium density, rather than absolute sodium intake, as the primary measure of sodium intake to investigate the association between dietary sodium and HT. This analysis is based on data from the 2007–2012 National Health and Nutrition Examination Survey (NHANES) and aims to provide evidence that could inform HT prevention strategies.

## 2 Methods

### 2.1 Study population

This cross-sectional study used data from the NHANES database provided by the U.S. Centers for Disease Control and Prevention (CDC). NHANES provides a comprehensive assessment of the health and nutritional status of a representative sample of the non-hospitalized US population. Detailed sampling methods are available on the NHANES website.^1^ The survey protocol was approved by the Ethics Review Committee of the National Center for Health Statistics (NCHS), with all participants providing written informed consent.^2^

**Figure 1:**
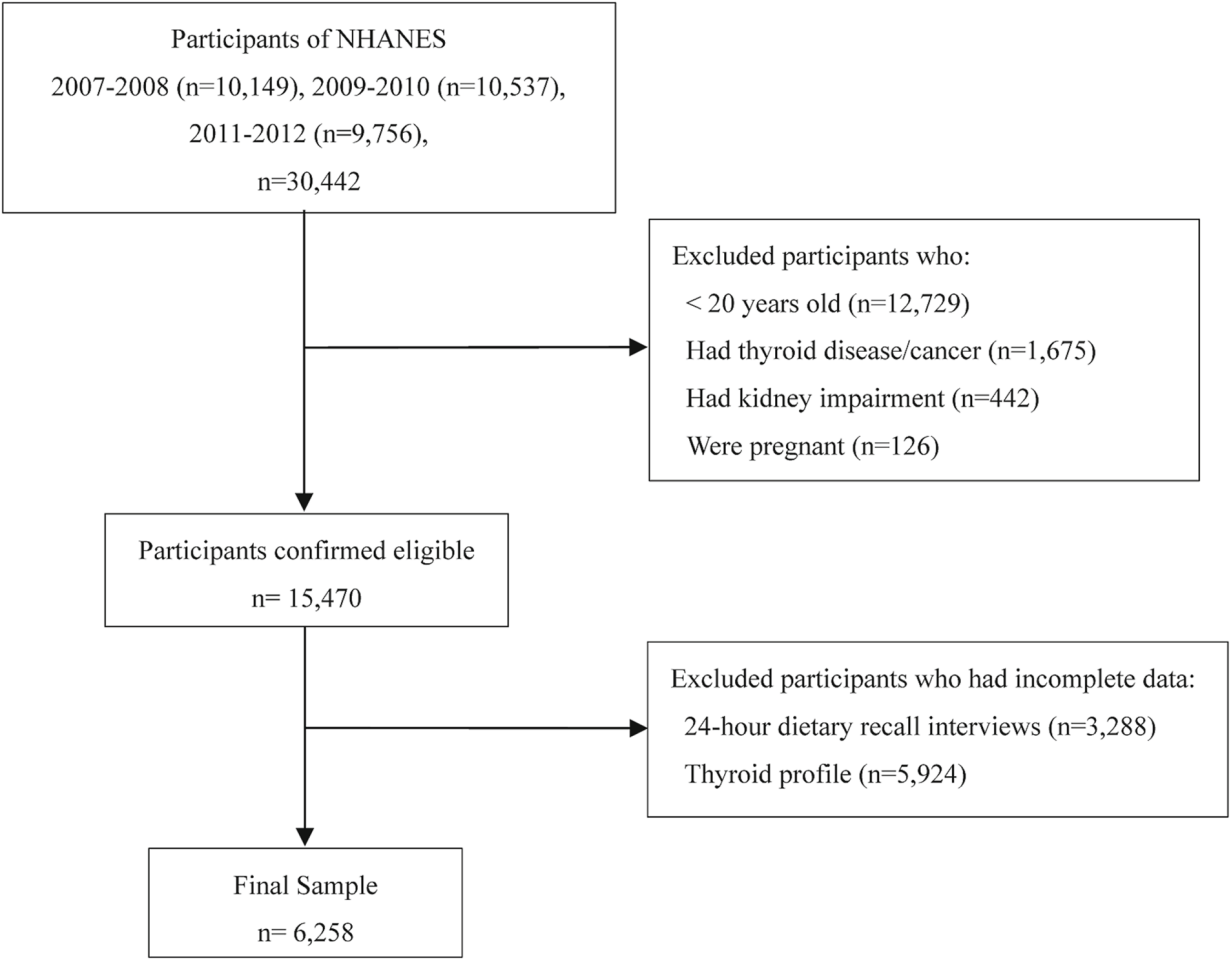
Participant flow diagram.

While dietary sodium and energy intake data are available across all NHANES datasets, complete thyroid profile data are only available in the 2007–2012 dataset (three two-year cycles). To ensure the validity and reliability of the analysis, we applied the following exclusion criteria: (1) participants under 20 years old (*n* = 12,729) due to inconsistent thyroid function data and developmental variability; (2) individuals with thyroid disease or cancer (*n* = 1,675) due to potential treatment-related bias; (3) those with kidney impairment (*n* = 442) due to effects on sodium metabolism; (4) pregnant participants (*n* = 126) because pregnancy alters thyroid function and immune responses; and (5) participants with incomplete dietary recall (*n* = 3,288) or thyroid profile data (*n* = 5,924). The final analysis included 6,258 participants, with the selection process detailed in Figure 1.

### 2.2 Diagnostic criteria

Serum thyroid hormone levels were measured as follows: Free triiodothyronine (FT3) was assessed using competitive binding immunoenzymatic assays; free thyroxine (FT4) via a two-step enzyme immunoassay; and thyroid-stimulating hormone (TSH) using a third-generation two-site immunoenzymatic assay. Thyroid peroxidase antibody (TPOAb) and thyroglobulin antibody (TgAb) titers were measured with the Beckman access2 immunoassay system. TPOAb positivity was defined as ≥9.0 IU/mL, and TgAb positivity as ≥115.0 IU/mL (16). HT was defined by positivity for either TPOAb or TgAb (1, 17).

### 2.3 Sodium density

Dietary intake was assessed through two 24-h recall interviews: the first in person at the Mobile Examination Center (MEC) and the second by telephone 3 to 10 days later. By using the U.S. Department of Agriculture’s Food and Nutrition Database for Dietary Studies, the daily total of all nutrients/food ingredients from all foods was calculated and entered into the NHANES database. Average sodium and energy intake were calculated from these two recalls, including diet and supplements. Sodium density, defined as milligrams of sodium per kilocalorie consumed, was used as the primary variable of interest.

### 2.4 Study covariates

To account for potential confounding, we included the following covariates: age, gender, race, education, marital status, income to poverty ratio (IPR), body mass index (BMI), history of hypertension, history of diabetes, history of rheumatoid arthritis, urinary iodine-creatinine ratio (UI/Cr), urinary albumin-creatinine ratio (UACR), energy intake, sleep disorder, smoking status, drinking status, and moderate activity. The study population was racially grouped as Mexican American, non-Hispanic White, other Hispanic, non-Hispanic Black, and others. UI/Cr and UACR were calculated using the formulas: UI/Cr (μg/g) = urinary iodine (μg/L) / urinary creatinine (mg/dL) × 100; UACR (mg/g) = urinary albumin (mg/L) / urinary creatinine (mg/dL) × 100. Smoking status was categorized as “smoker” (more than 100 cigarettes in a lifetime) or “non-smoker,” and drinking status as “drinker” (at least five glasses of alcohol in the past 12 months) or “non-drinker.” Sleep disorders were self-reported in response to the question, “Have you/Has S*P* ever been told by a doctor or other health professional that you have a sleep disorder?” Moderate activity was self-reported in response to the question, “Do you/Does S*P* do any moderate-intensity sports, fitness, or recreational activities that cause a small increase in breathing or heart rate such as brisk walking, bicycling, swimming, or golf for at least 10 min continuously?”

### 2.5 Statistical analysis

Data extraction and merging for the 2007–2012 NHANES data were performed using R Studio (version 4.4.1). All data analyses and figure generation were conducted using SPSS software (version 22.0) and R Studio (version 4.4.1).

The data distribution was assessed for normality using the Shapiro–Wilk test, which indicated deviations from normality for several variables. As a result, non-parametric tests, such as the Mann–Whitney U test and the Chi-square test, were used to compare continuous and categorical variables, respectively.

The data analysis followed a logical and chronological sequence. Initially, K-means clustering was performed to categorize sodium density into three distinct classes: Class 1 (<1.504 mg/kcal), Class 2 (1.504 mg/kcal–2.076 mg/kcal), Class 3 (>2.076 mg/kcal). The optimal number of clusters was determined using the elbow method (Supplementary Figure S1). This classification informed the subsequent multivariate logistic regression analyses, which examined associations between sodium density and HT, TPOAb positivity, and TgAb positivity. Restricted cubic spline (RCS) modeling was then applied to explore potential non-linear relationships. Based on the RCS results, a recursive algorithm was used to identify a breakpoint in the relationship between sodium density and HT risk. Finally, two-segment generalized linear regression modeling was performed to further characterize this relationship.

The correlations between sodium density and other characteristics were assessed using the Spearman correlation coefficient and presented in a heatmap. We assessed the interaction effect by including interaction terms (sodium density * age/gender/race/education/marital status/IPR/diabetes/hypertension/rheumatoid arthritis/BMI/UI/Cr/UACR/smoking status/drinking status/sleep disorder/moderate activity) in the logistic regression model.

## 3 Results

### 3.1 Characteristics of participants

This study included 6,258 participants, with 3,285 males and 2,973 females, aged 20 to 80 years. Of these, 576 individuals (9.2%) were diagnosed with HT. Younger participants, males, and non-Hispanic Black individuals had lower rate of HT (*p* < 0.05). Patients with HT had higher UI/Cr, UACR, and TSH levels, and lower levels of FT3, FT4, energy intake, and sodium intake compared to those without HT (*p* < 0.05). Moreover, they were more likely to be widowed, separated, or divorced. While the median sodium density was slightly higher in HT patients (1.64 mg/kcal vs. 1.61 mg/kcal, *p* = 0.043), this small difference in sodium density becomes more pronounced when transformed to absolute sodium intake, such as 3,280 mg/d versus 3,220 mg/d at the 2,000-kilocalorie level. Other characteristics did not differ significantly between the two groups (*p* > 0.05). The characteristics of all participants, including those with and without HT, are detailed in Table 1.

**Table 1:** Characteristics of selected participants from the NHANES 2007–2012.

Characteristic	Total *n* = 6,258	Non-Hashimoto’s thyroiditis *n* = 5,682 (90.80%)	Hashimoto’s thyroiditis *n* = 576 (9.20%)	*p* value
Age, years	49.00 (35.00–63.00)	48.00 (34.00–63.00)	54.00 (40.00–67.00)	<0.001
Gender (%)				<0.001
Male	3,285 (52.49)	3,053 (53.73)	232 (40.28)	
Female	2,973 (47.51)	2,629 (46.27)	344 (59.72)	
Race (%)				<0.001
Mexican American	999 (15.96)	899 (18.52)	100 (17.36)	
Other Hispanic	671 (10.72)	603 (10.61)	68 (11.81)	
Non-Hispanic White	2,895 (46.26)	2,592 (45.62)	303 (52.60)	
Non-Hispanic Black	1,299 (20.76)	1,232 (21.68)	67 (11.63)	
Others	394 (6.30)	356 (6.27)	38 (6.60)	
Education level (%)				0.894
<High school	1725 (27.56)	1,571 (27.65)	154 (26.74)	
High school	1,469 (23.47)	1,333 (23.46)	136 (23.61)	
>High school	3,064 (48.96)	2,778 (48.89)	286 (49.65)	
Marital status (%)				0.001
Married or living with partner	3,821 (61.06)	3,464 (60.96)	357 (61.98)	
Widowed/separated/divorced	1,339 (21.40)	1,193 (21.00)	146 (25.35)	
Never married	1,098 (17.55)	1,025 (18.04)	73 (12.67)	
IPR (%)				0.243
<5.00	6,143 (98.16)	5,574 (98.10)	569 (98.78)	
≥5.00	115 (1.84)	108 (1.90)	7 (1.22)	
Smoking status (%)				0.136
Smoker	2,890 (46.18)	2,641 (46.48)	249 (43.23)	
Non-smoker	3,368 (53.82)	3,041 (53.52)	327 (56.77)	
Drinking status (%)				0.183
Drinker	4,325 (69.11)	3,941 (69.36)	384 (66.67)	
Non-drinker	1933 (30.89)	1741 (30.64)	192 (33.33)	
Sleep disorder (%)				0.154
Yes	461 (7.37)	427 (7.51)	34 (5.90)	
No	5,797 (92.63)	5,255 (92.49)	542 (94.10)	
Moderate activity (%)				0.918
Yes	2,470 (39.47)	2,241 (39.44)	229 (39.76)	
No	3,788 (60.53)	3,441 (60.56)	347 (60.24)	
Diabetes (%)				0.861
Yes	699 (11.17)	631 (11.10)	68 (11.80)	
Borderline	103 (1.65)	93 (1.64)	10 (1.74)	
No	5,456 (87.18)	4,958 (87.26)	498 (86.46)	
Hypertension (%)				0.605
Yes	2069 (33.06)	1873 (32.96)	196 (34.03)	
No	4,189 (66.94)	3,809 (67.04)	380 (65.97)	
Rheumatoid arthritis (%)				0.462
Yes	369 (5.90)	339 (5.97)	30 (5.21)	
No	5,889 (94.10)	5,343 (94.03)	546 (94.79)	
BMI, kg/m^2^	27.90 (24.40–32.09)	27.90 (24.40–32.15)	27.61 (24.41–31.60)	0.473
UI/Cr, μg/g	141.76 (87.88–235.29)	141.76 (86.75–232.32)	159.06 (99.35–267.59)	<0.001
UACR, mg/g	6.80 (4.44–12.77)	6.80 (4.40–12.59)	7.21 (4.91–15.26)	0.001
FT3, pg/mL	3.13 (2.90–3.40)	3.16 (2.90–3.40)	3.10 (2.88–3.30)	<0.001
FT4, ng/dL	10.30 (9.00–11.50)	10.30 (9.00–11.50)	9.90 (9.00–11.03)	<0.001
TSH, μIU/mL	1.54 (1.05–2.31)	1.51 (1.03–2.21)	2.16 (1.38–3.34)	<0.001
Energy intake, kcal/d	1919.00 (1461.88–2482.63)	1930.50 (1475.00–2496.12)	1813.25 (1374.62–2370.25)	<0.001
Sodium intake, mg/d	3097.00 (2300.88–4098.88)	3111.00 (2310.62–4109.50)	2913.25 (2181.75–3953.38)	0.010
Sodium density, mg/kcal	1.61 (1.37–1.90)	1.61 (1.36–1.90)	1.64 (1.40–1.94)	0.043

Data were the actual number of participants (weighted percentage) or medians (interquartile ranges). IPR, income to poverty ratio; BMI, body mass index; UI/Cr, urinary iodine-creatinine ratio; UACR, urinary albumin-creatinine ratio; FT3, free triiodothyronine; FT4, free thyroxine; TSH, thyroid-stimulating hormone; TgAb, thyroglobulin antibodies; TPOAb, thyroid peroxidase antibodies.

### 3.2 Association between sodium density and HT

Multivariate logistic regression models revealed that, for each additional mg/kcal of sodium density, the risk of HT increased by 22 to 25% across models (Model 1: odds ratio [OR] 1.22, 95% confidence interval [CI] 1.01–1.48, *p* = 0.044; Model 2: OR 1.25, 95% CI 1.02–1.52, *p* = 0.030; Model 3: OR 1.24, 95% CI 1.01–1.50, *p* = 0.037) (Table 2). Participants in the highest sodium density class (Class 3) were significantly more likely to have HT compared to those in the lowest class (Class 1) across models (Model 1: OR 1.37, 95% CI 1.07–1.76, *p* = 0.013; Model 2: OR 1.42, 95% CI 1.10–1.83, *p* = 0.007; Model 3: OR 1.41, 95% CI 1.09–1.82, *p* = 0.009), with *P*-trend <0.05 (Table 2).

**Table 2:** The association between dietary sodium density and HT.

Exposure	Model 1		Model 2		Model 3	
	OR (95% Cl)	*p* value	OR (95% Cl)	*p* value	OR (95% Cl)	*p* value
Sodium density (mg/kcal)	1.22 (1.01–1.48)	0.044	1.25 (1.02–1.52)	0.030	1.24 (1.01–1.50)	0.037
Class 1	Reference		Reference		Reference	
Class 2	1.07 (0.88–1.29)	0.513	1.08 (0.89–1.31)	0.421	1.08 (0.89–1.31)	0.448
Class 3	1.37 (1.07–1.76)	0.013	1.42 (1.10–1.83)	0.007	1.41 (1.09–1.82)	0.009
*P*-trend	0.045		0.029		0.034	

Model 1: age, gender, and race were adjusted. Model 2: additionally adjusted for body mass index, diabetes, hypertension, rheumatoid arthritis, urinary iodine-creatinine ratio, urinary albumin-creatinine ratio, energy intake. Model 3: additionally adjusted for education, marital status, income to poverty ratio, smoking status, drinking status, sleep disorder and moderate activity. OR: odds ratio; CI: confidence interval.

### 3.3 Non-linear relationship between sodium density and HT

**Figure 2:**
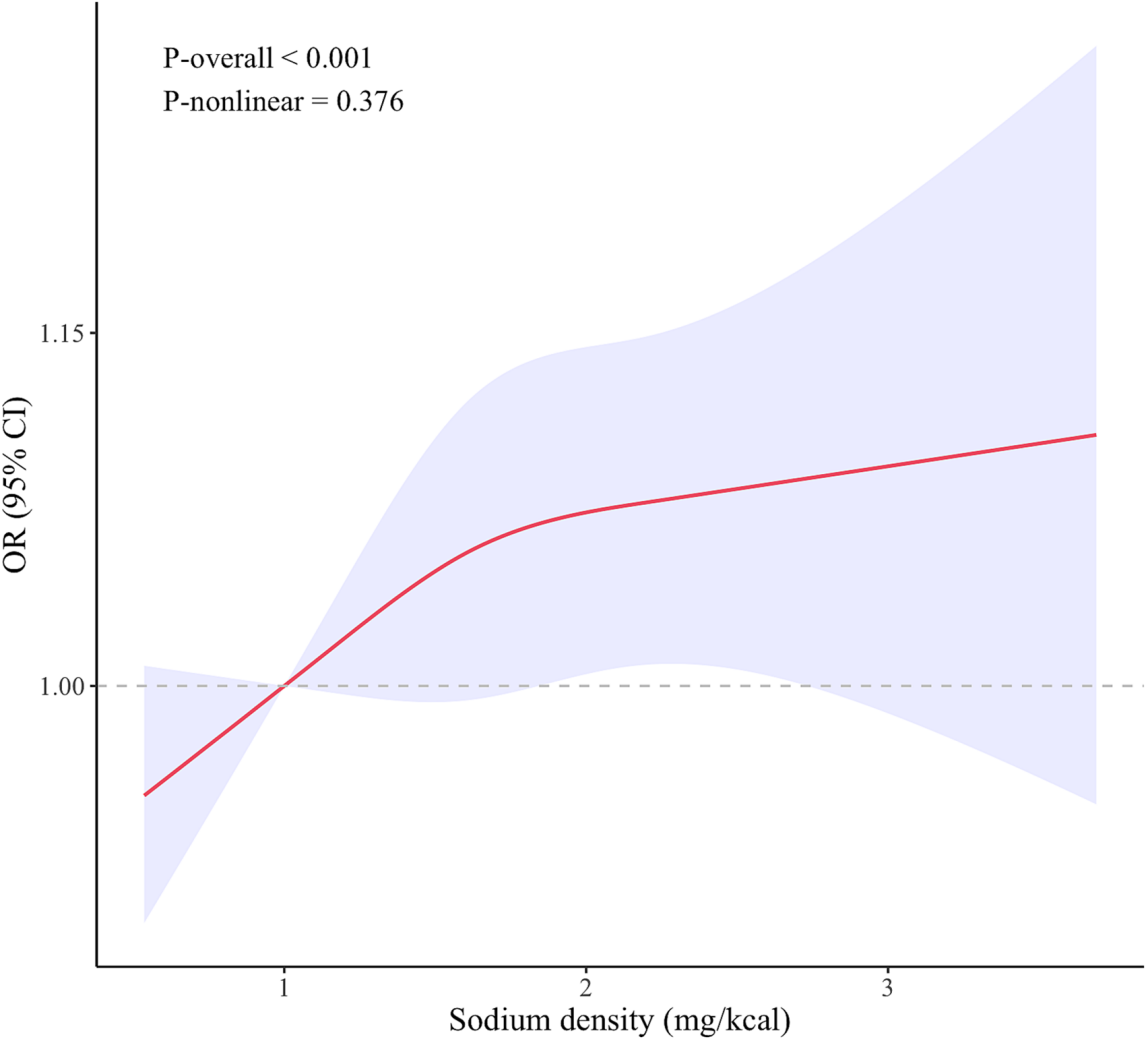
Restricted cubic spline model for the relationship between dietary sodium density and HT. The 95% CIs of the adjusted ORs were represented by the blue-shaded area. The model was adjusted for all covariates. OR, odds ratio; CI, confidence interval.

To further explore the relationship between sodium density and HT risk, we analyzed potential non-linear associations using RCS (Figure 2). The results did not reveal statistically significant non-linearity (*P*-nonlinear = 0.376). However, using a two-segment generalized linear model and recursive algorithm, we identified a breakpoint at 2.43 mg/kcal (Table 3). Below this threshold, there was a positive correlation between sodium density and HT risk (adjusted *β* 0.30, 95% CI 0.05–0.56, *p* = 0.019), but this correlation was not existent when sodium density exceeded 2.43 mg/kcal. This highlights a potential non-linear relationship between sodium density and HT risk.

**Table 3:** Threshold effect analysis of dietary sodium density on HT using two-piecewise generalized linear regression.

Model	Adjusted *β* (95%Cl)	*p* value
Fitting by standard linear model	0.21 (0.01–0.41)	0.036
Fitting by two-piecewise linear model breakpoint (mg/kcal)	2.43	
< 2.43 mg/kcal	0.30 (0.05–0.56)	0.019
> 2.43 mg/kcal	−0.67 (−1.89–0.55)	0.287
Likelihood ratio test		0.391

The models were adjusted for all covariates. CI, confidence interval.

### 3.4 Association between sodium density and TPOAb/TgAb positivity

Given that HT is diagnosed on the basis of the presence of antibodies in the thyroid gland, we also examined the relationship between dietary sodium density and TPOAb/TgAb positivity (Table 4). In all models, a 1 mg/kcal increase in sodium density was associated with a 26 to 29% higher risk of TPOAb positivity (Model 1: OR 1.26, 95% CI 1.04–1.53, *p* = 0.021; Model 2: OR 1.29, 95% CI 1.06, 1.57; *p* = 0.013; Model 3: OR 1.28, 95% CI 1.05–1.56, *p* = 0.016). Additionally, participants in the highest sodium density class (Class 3) had significantly higher rate of TPOAb positivity compared to those in the lowest class (Class 1) across models (Model 1: OR 1.42, 95% CI 1.10–1.83, *p* = 0.007; Model 2: OR 1.47, 95% CI 1.13–1.90, *p* = 0.004; Model 3: OR 1.46, 95% CI 1.12–1.89, *p* = 0.004), with *P*-trend <0.05. However, sodium density was not associated with TgAb positivity in any model (*p* > 0.05).

**Table 4:** The association between dietary sodium density and TPOAb positive, TgAb positive.

Exposure	Model 1		Model 2		Model 3	
	OR (95% Cl)	*p* value	OR (95% Cl)	*p* value	OR (95% Cl)	*p* value
TPOAb positivity						
Sodium density (mg/kcal)	1.26 (1.04–1.53)	0.021	1.29 (1.06–1.57)	0.013	1.28 (1.05–1.56)	0.016
Class 1	Reference		Reference		Reference	
Class 2	1.11 (0.91–1.35)	0.313	1.12 (0.92–1.37)	0.242	1.12 (0.92–1.37)	0.256
Class 3	1.42 (1.10–1.83)	0.007	1.47 (1.13–1.90)	0.004	1.46 (1.12–1.89)	0.004
*P*-trend	0.029		0.016		0.020	
TgAb positivity						
Sodium density (mg/kcal)	0.96 (0.53–1.67)	0.891	0.99 (0.54–1.74)	0.961	0.99 (0.54–1.74)	0.963
Class 1	Reference		Reference		Reference	
Class 2	0.93 (0.54–1.63)	0.806	0.95 (0.54–1.67)	0.857	0.95 (0.54–1.67)	0.857
Class 3	1.05 (0.50–2.21)	0.900	1.08 (0.48–2.25)	0.838	1.09 (0.49–2.27)	0.831
*P*-trend	0.963		0.932		0.912	

Model 1: age, gender, and race were adjusted. Model 2: additionally adjusted for body mass index, diabetes, hypertension, rheumatoid arthritis, urinary iodine-creatinine ratio, urinary albumin-creatinine ratio, energy intake. Model 3: additionally adjusted for education, marital status, income to poverty ratio, smoking status, drinking status, sleep disorder and moderate activity. OR: odds ratio; CI: confidence interval.

### 3.5 Non-linear relationship between sodium density and TPOAb/TgAb positivity

**Figure 3:**
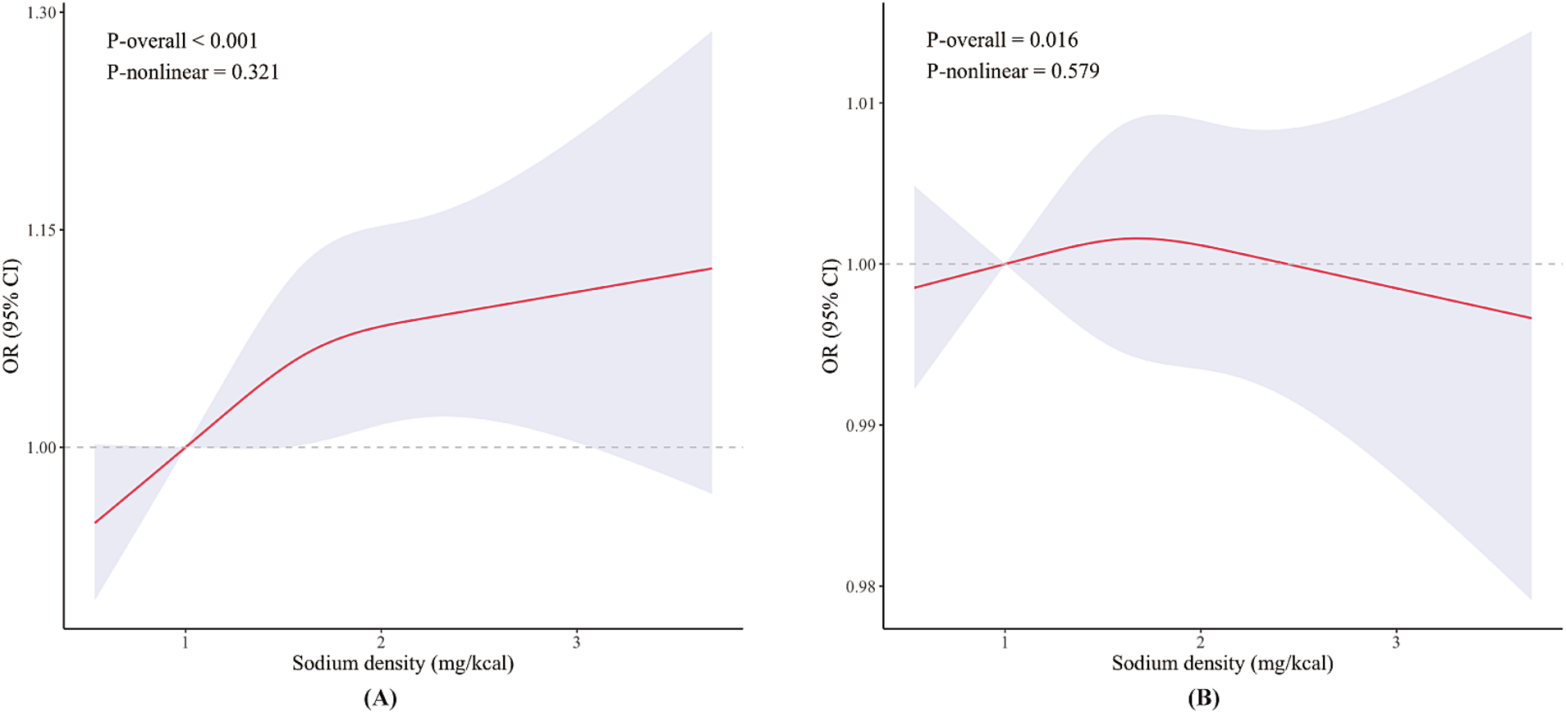
Restricted cubic spline models for the relationship of dietary sodium density with TPOAb positivity **(A)** and TgAb positivity **(B)**. The 95% CIs of the adjusted ORs were represented by the blue-shaded area. Both models were adjusted for all covariates. OR, odds ratio; CI, confidence interval.

Subsequently, we further analyzed the non-linear relationship between sodium density and TPOAb/TgAb positivity using RCS (Figure 3). Similar to HT risk, the association between sodium density and TPOAb positivity followed a nearly inverted L-shape, though this relationship was not statistically significant (*P*-overall <0.001, *P*-nonlinear = 0.321). Consistent with earlier findings, sodium density was not associated with TgAb positivity (*P*-overall = 0.016; *P*-nonlinear = 0.579).

### 3.6 Associations between sodium density and other characteristics

**Figure 4:**
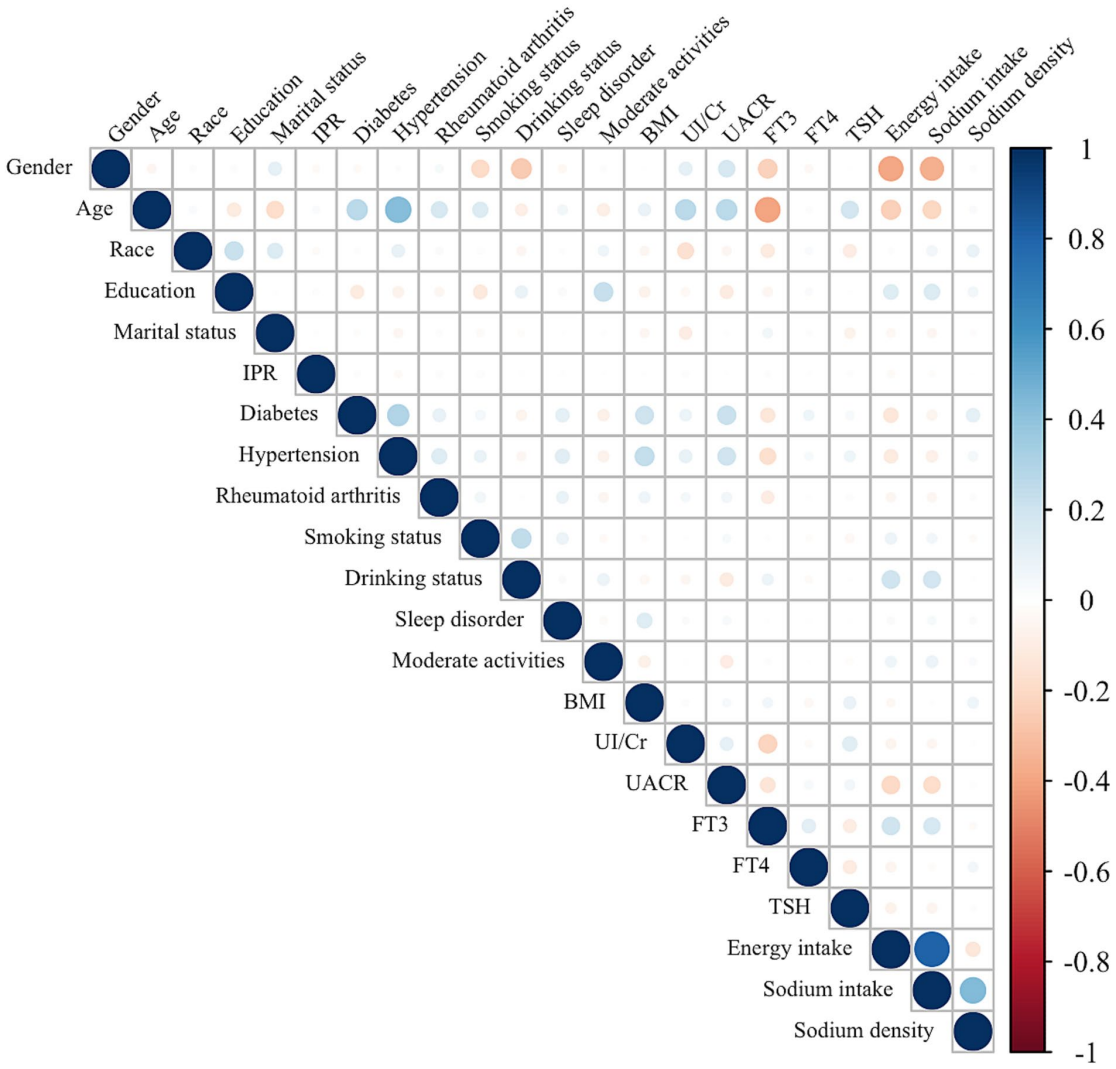
Correlation between the dietary sodium density and other characteristics in whole study population. The color represents the association’s strength. The Spearman correlation coefficient was used to calculate the correlation.

The associations between dietary sodium density and other characteristics were evaluated and presented in Figure 4. Sodium density was positively correlated with race (*r* = 0.09, *p* < 0.001), education level (*r* = 0.05, *p* < 0.001), BMI (*r* = 0.07, *p* < 0.001), diabetes (*r* = 0.11, *p* < 0.001), hypertension (*r* = 0.05 *p* < 0.001), FT4 (*r* = 0.05, *p* < 0.001), and sodium intake (*r* = 0.43, *p* < 0.001). Conversely, it was negatively correlated with energy intake (*r* = −0.12, *p* < 0.001). It should be noticed that there was a strong correlation between energy intake and sodium intake (*r* = 0.81, *p* < 0.001), similar to previous reports (15), and the correlations of energy intake and sodium intake with other characteristics were almost identical. Although correlations between sodium density and race, BMI, education level, hypertension, or FT4 levels were statistically significant, the small coefficients (*r* < 0.1) suggest limited clinical relevance. Nevertheless, the potential interaction between sodium intake, BMI, and thyroid function highlights areas for future exploration to better understand the pathogenesis of HT.

### 3.7 Interaction of sodium density and other covariates

We tested for potential interaction effects between sodium density and various covariates to assess whether these factors influenced the relationship between sodium density and HT risk. As shown in Supplementary Table S1, no significant interactions were found between sodium density and age, gender, race, education, marital status, IPR, BMI, diabetes, hypertension, rheumatoid arthritis, or other factors (*P*-interaction >0.05 for each).

## 4 Discussion

This large retrospective study explored the potential relationship between dietary sodium density and HT by merging and analyzing NHANES data from 2007 to 2012. To our knowledge, this is the first cross-sectional analysis to assess the association between dietary sodium intake and HT. Our results indicate that dietary sodium density is positively correlated with HT risk, with each additional mg/kcal of sodium density increasing the risk by 24% (OR 1.24, 95% CI 1.01–1.50, *p* = 0.037). The relationship between sodium density and HT risk appeared to be nearly non-linear based on RCS analysis and a two-segment linear model, with a breakpoint at 2.43 mg/kcal. This suggests that increased dietary sodium density elevates the risk of HT before the breakpoint. Although the risk of HT does not continue to rise consistently beyond this point, it remains very high. Additionally, per 1 mg/kcal increase in sodium density was associated with a 28% increased risk of TPOAb positivity (OR 1.28, 95% CI 1.05–1.56, *p* = 0.016).

Excessive sodium consumption is a well-established risk factor for increased mortality and cardiovascular diseases. Recent research has uncovered new links between sodium intake and health, particularly its pro-inflammatory effects. High sodium intake can stimulate certain immune system components, enhancing the body’s ability to combat pathogens but also provoking stronger autoimmune responses (10). Animal studies consistently demonstrate that high sodium intake exacerbates autoimmune conditions by reversing the suppressive effects of Tregs and promoting a shift toward Th-1 and Th17 pro-inflammatory phenotypes, which secrete inflammatory mediators (3, 11, 12, 18). Unfortunately, the current literature lacks *in vivo* studies investigating the relationship between sodium intake and HT. In terms of thyroid cell injury, cytokines derived from the lymphocytic infiltrate play a key role, including their capacity to stimulate the thyroid cells themselves to release proinflammatory mediators, thereby amplifying and perpetuating the autoimmune response (19). Clinical and epidemiological studies, although scarce, are in accord with these findings supporting the notion that high sodium intake is a potential environmental trigger for autoimmune diseases such as rheumatoid arthritis and SLE. Two cross-sectional studies reported a significant association between higher sodium intake and rheumatoid arthritis, particularly among smokers (20, 21). Correa-Rodríguez et al. (22) founded that dietary sodium intake was significantly associated with anti-dsDNA and complement C4 level, indicating that sodium intake may play a key role in markers related to disease activity in SLE patients. A clinical trial involving patients with rheumatoid arthritis and SLE suggested that a low-sodium diet could reduce the inflammatory response by decreasing the percentage of Th17 cells (23). We did not find any study that directly explored the relationship between HT and sodium intake, but two studies evaluating the intake frequencies of food groups showed that patients with HT consumed more high-salted processed meat than controls (6, 7).

Emerging research highlights that microbiota alterations could be closely related to the development and progression of HT, with the most significant correlations reported for TPOAb (24). High dietary sodium intake can disrupt gut microbiota, particularly by depleting *Lactobacillus murinus*, which reduces the production of short-chain fatty acids that inhibit Th17 conversion, thereby affecting gut immune homeostasis and further contributing to an increase in Th17 cells (25). In addition, high sodium level can interfere with iodine absorption (26), a vital nutrient for thyroid hormone synthesis (27, 28), thereby worsening autoimmune thyroid conditions. These potential pathogenic mechanisms are in alignment with the findings of this study, indicating that high sodium intake is a risk factor for the development of HT.

TPOAb and TgAb are key autoantibodies involved in AITD, but they play different roles and exhibit distinct diagnostic significance in HT (1, 17). TPOAb, present in approximately 95% of HT patients, is widely recognized as the most reliable biomarker of HT (1, 29). Thyroid peroxidase, the target of TPOAb, is essential for thyroid hormone synthesis, directly influencing the production of T4 and T3 (1). In contrast, TgAb, which targets thyroglobulin (a storage protein for thyroid hormones), is present in 60–80% of HT cases and is less consistent as a diagnostic marker (1). In our study, the strong association observed between sodium intake and TPOAb positivity, but not TgAb, aligns with these differences in their roles. Among HT subjects in our cohort, 96.5% were positive for TPOAb, while only 10.6% were positive for TgAb. This significant disparity suggests that TgAb may represent an earlier or more transient immune response, while TPOAb reflects a later, more established phase of immune activation, possibly indicative of immune escalation (3). This immunological distinction helps explain why dietary sodium intake was associated with TPOAb positivity but not TgAb. Sodium’s known pro-inflammatory effects, such as shifting the immune balance toward Th17-mediated responses (3, 11, 18), may exacerbate the production of TPOAb during the progressive stages of autoimmunity. TgAb, being less prevalent and potentially tied to earlier immune events, may not exhibit the same correlation with environmental triggers like sodium intake.

The association between iodine intake and the presence of circulating thyroid antibodies is complex, with iodine intake both below and above the recommended level being associated with an increase in circulating antibodies (27, 28). In this study, iodine intake was considered by including UI/Cr, a reliable indicator of iodine nutritional status, in the regression analysis as a confounding variable. We also excluded patients with renal impairment and included the UACR as a confounding factor to eliminate differences in renal sodium excretion among participants. Significant differences in UACR levels were observed between HT patients and non-HT individuals, likely due to correlations of UACR with gender (*r* = 0.17, *p* < 0.001) and age (*r* = 0.27, *p* < 0.001). Differences in absolute sodium intake and energy intake between the two groups could also be explained in this way. Bewilderingly, while rheumatoid arthritis, smoking, alcohol consumption, sleep disorders, and physical inactivity have been reported as possible risk factors for AITD (4, 30, 31), this study found no significant associations between these factors and HT.

Healthy dietary patterns typically limit sodium intake to the Chronic Disease Risk Reduction (CDRR) level defined by the U.S. National Academy of Sciences, which recommends a maximum of 2,300 mg/day for individuals aged 13 years and older (32). However, according to the WHO’s *Global Report on Sodium Intake Reduction*, the global average sodium intake is estimated to be 4,310 mg/day—more than twice the recommended level of 2,000 mg/day (33). Increasing evidence has linked high sodium intake to various adverse health outcomes, including gastric cancer, obesity, osteoporosis, and kidney disease (33). In our study, using a recursive algorithm and a two-segment generalized linear model, we identified a sodium density breakpoint of 2.43 mg/kcal (equivalent to 4,860 mg/day of sodium or 12.15 g/day of salt at a 2,000-kcal level). Below this threshold, sodium density was positively associated with an increased risk of HT. These findings not only highlight a potential dietary threshold for sodium in relation to HT risk but also align with broader public health efforts to reduce excessive sodium intake.

Currently, most clinical and epidemiological studies focus on the relationship between AITD and iodized salt (iodine) consumption, with no research on dietary sodium consumption and HT. Our study, conducted from a population perspective, provides evidence for a potential association between higher dietary sodium intake and increased prevalence of HT and TPOAb positivity. However, this study has several limitations. First, dietary sodium density was assessed using two 24-h recalls, which may introduce recall bias and may not accurately reflect usual sodium intake, though some studies suggest that two 24-h recalls can adequately estimate daily dietary intake (34). Second, although serum anti-thyroid antigen antibodies are the gold standard for diagnosing HT, some clinical features, ultrasound manifestations, and other related indicators can be considered. These factors could not be included in the study because there was no relevant data in the NHANES database. Third, this study only analyzed sodium intake without exploring the relationship between other nutrients and HT. Such as dietary selenium, Iron and vitamin D, etc. (27, 35), these may play an important role in the occurrence and development of HT. Finally, as this is a cross-sectional study, the findings demonstrate associations rather than causations. Prospective or interventional studies are required to confirm the causal relationships between dietary sodium density and HT.

## 5 Conclusion

In this study, we found that the prevalence of HT increases with increasing dietary sodium density and that dietary sodium density is independently and positively associated with TPOAb positivity. These findings provide new evidence supporting dietary sodium reduction as a potential strategy for HT prevention. Future studies are needed to better understand the underlying mechanisms and to inform the development of dietary guidelines aimed at mitigating HT risk.

## Data Availability

Publicly available datasets were analyzed in this study. This data can be found here: https://www.cdc.gov/nchs/nhanes/nhanes.

## Footnotes

^1^ https://www.cdc.gov/nchs/nhanes/index.htm

^2^ https://www.cdc.gov/nchs/nhanes/irba98.htm
