# Supplementary material for: The association between dietary sodium density and Hashimoto’s thyroiditis in US adults
Source: Front Nutr. 2025 Jan 22;12:1508195. doi: 10.3389/fnut.2025.1508195 (PMC11794099; doi:10.3389/fnut.2025.1508195)
Supplement: Supplementary file 1 [file Data_Sheet_1.docx]

Supplementary Material

# Supplementary Figures and Tables

## Supplementary Figures

##
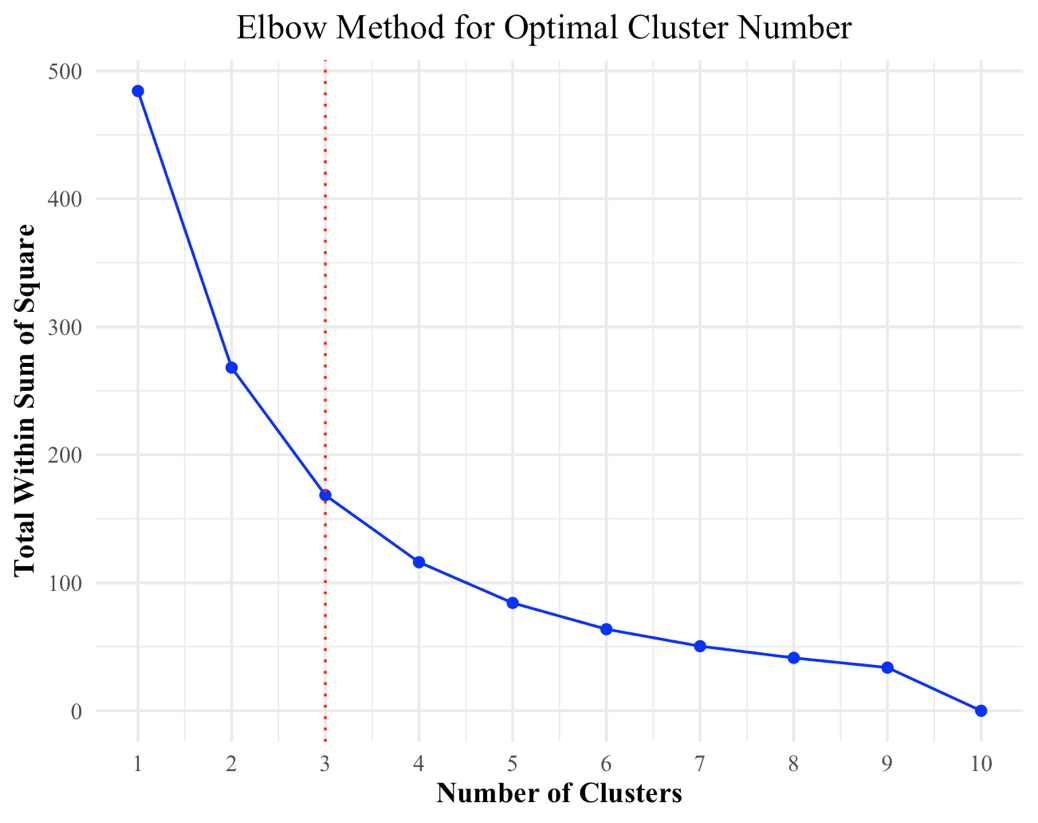


## Figure S1: Elbow method to identify the appropriate number of clusters.

## Supplementary Tables

**S-Table 1** Interaction analysis of dietary sodium density with other covariate on TPOAb positivity

| Interaction | OR (95 % CI) | *P*-interaction |
| --- | --- | --- |
| Sodium density ^ Age | 1.00 (0.99, 1.01) | 0.817 |
| Sodium density ^ Gender | 0.84 (0.57, 1.25) | 0.393 |
| Sodium density ^ Race | 1.05 (0.90, 1.24) | 0.515 |
| Sodium density ^ Education | 1.01 (0.88, 1.27) | 0.940 |
| Sodium density ^ Marital status | 0.87 (0.66, 1.28) | 0.301 |
| Sodium density ^ IPR | 0.16 (0.01, 1.57) | 0.148 |
| Sodium density ^ BMI | 0.99 (0.96, 1.02) | 0.582 |
| Sodium density ^ Diabetes | 1.03 (0.68, 1.56) | 0.874 |
| Sodium density ^ Hypertension | 1.10 (0.73, 1.64) | 0.645 |
| Sodium density ^ Rheumatoid arthritis | 1.51 (0.67, 3.27) | 0.300 |
| Sodium density ^ UI/Cr | 1.00 (1.00, 1.00) ^1^ | 0.482 |
| Sodium density ^ UACR | 1.00 (1.00, 1.00) ^1^ | 0.235 |
| Sodium density ^ Smoking status | 1.40 (0.94, 2.08) | 0.095 |
| Sodium density ^ Drinking status | 1.26 (0.84, 1.92) | 0.274 |
| Sodium density ^ Sleep disorder | 1.11 (0.51, 2.33) | 0.789 |
| Sodium density ^ Moderate activity | 0.97 (0.65, 1.44) | 0.868 |

The models were adjusted for all covariates.

^1^ It typically indicates that during the model fitting process, the predicted probabilities for these observations are numerically very close to 1.
